# Selecting Dogs for Explosives Detection: Behavioral Characteristics

**DOI:** 10.3389/fvets.2020.00597

**Published:** 2020-09-02

**Authors:** Lucia Lazarowski, Lowell Paul Waggoner, Sarah Krichbaum, Melissa Singletary, Pamela Haney, Bart Rogers, Craig Angle

**Affiliations:** ^1^Canine Performance Sciences Program, College of Veterinary Medicine, Auburn University, Auburn, AL, United States; ^2^Department of Psychology, College of Liberal Arts, Auburn University, Auburn, AL, United States; ^3^Department of Anatomy, Physiology, and Pharmacology, College of Veterinary Medicine, Auburn University, Auburn, AL, United States

**Keywords:** detection dogs, detection dog evaluation, explosives detection dogs, working dogs, selection, canine

## Abstract

Detection dogs are widely considered the most effective and adaptive method for explosives detection. Increases in emerging sophisticated threats are accelerating the demand for highly capable explosives detection, causing a strain on available supplies of quality canines worldwide. These strains are further compounded by rigorous behavioral standards required to meet mission-specific capabilities, leading to high rates of dogs disqualified from training or deployment. Ample research has explored the behavioral characteristics important for assistance, guide, and other traditional working roles, while those corresponding to more specialized tasks such as detection of explosives are not as well-understood. In this review we aim to identify the behavioral characteristics important for operational tasks of explosives detection dogs, contrasting with that of other working roles and highlighting key differences between explosives and other types of detection dogs. Further, we review the available research on methods for assessing and selecting candidate detection dogs and make recommendations for future directions and applications to the industry. Improvements and standardization in assessment technology allowing for the identification and enhancement of behavioral characteristics will be key to advancing canine detection technology in general.

## Introduction

Increasing recognition of the detection dog as the most capable and adaptable method for real-time detection of explosives has led to a world-wide increase in their use in security and military operations, which is straining the supply of dogs capable of performing explosives detection (1). The U.S. Congress has stated that U.S. dependence on foreign procurement and a lack of domestic production of explosive detection dogs (EDD) presents a critical security gap [(2), 115^th^ U.S. Congress]. Military and security officials from numerous nations attending the 2019 *International Working Dog Conference* of the *International Working Dog Breeding Association* noted the dwindling supply of suitable candidate EDDs from traditional private sources. Moreover, EDD tasks are increasingly specialized and sophisticated, further constraining the availability of dogs with the behavioral, physiological, and structural characteristics necessary to perform those tasks.

EDDs are primarily sourced from populations of dogs that have been selectively bred for hundreds of years for hunting, herding, and protection (3, 4). Substance detection tasks, which mostly occur in the context of intense human activity such as urban landscapes, are a relatively recent application of dogs for which there has been very limited directed selective breeding. Increasing evidence indicates that behavioral characteristics have a greater influence on detection dog success than sensory or morphological differences (5, 6). However, standardized and reliable methods for identifying suitable candidates are lacking, resulting in low rates of dogs achieving operational status and high levels of “behavioral wastage,” which has obvious implications for program efficiency as well as concerns regarding animal welfare (7). Given the extensive time involved and economic investment in the preparation of a working dog, as well as a lack of reliable predictors of success, identification and valid measurement of the expression of behavioral characteristics important to EDD performance are essential for accurate selection and, especially, the purpose-breeding of potential EDDs (7, 8) Therefore, better defining and communicating of the key behavioral characteristics of successful EDDs is critical to enhancing the supply of dogs capable of performing contemporary EDD tasks.

While characteristics have been fairly well-defined by research for assistance, guide, and some other working dogs, information about EDD characteristics is largely siloed within and varied across programs and has not been subject to much scientific examination and validation. Recent reviews have examined detection dog characteristics for wildlife/conservation dogs (3, 9), but there have been few systematic examinations and standardization of behavioral characteristics important to EDD performance (10, 11). In this review, we aim to identify behavioral characteristics that by general consensus, our experience in breeding and preparing dogs for explosives detection tasks over the last 20+ years, and pertinent research are important to EDD performance. We also review research on available methods for assessing and identifying candidate detection dogs and make recommendations for future directions and applications to the industry.

## Types of Explosives Detection Dogs

EDD is a general vocation defined by the class of targets to be detected (explosives) that implies some, but not all or even the predominant, capabilities necessary for performing the range of different EDD tasks. EDD tasks are more specifically defined by the parameters of the context and details of the search task. In many cases, the characteristics necessary for these varying EDD search tasks are the same but may vary significantly in the needed degree of expression. Traditionally, the most general EDD (sometimes referred to as “standard” EDD) is a dog that searches an array of areas at the immediate direction of a handler, most often but not exclusively on lead. Such areas include the interior and exterior of varied types of buildings, road vehicles, limited open areas (e.g., a park), and articles such as luggage and boxed goods. More specialized EDD applications are extensions of these general tasks and are often more focused on a particular search task, the context in which that search occurs, or the mode by which that search is performed.

As mentioned, specialization is increasing as a consequence of the growing sophistication of EDD applications with some fairly well-defined specialties. For example, Person-Borne Improvised Explosive Device (PBIED) EDDs interrogate persons or their aerodynamic wake, such as TSA *Passenger Screening* and *Vapor Wake*^®^ canines, respectively. *Specialized Search Dogs* (SSD) and similar variants are remotely-directed dogs working off-lead and down-range, primarily but not exclusively for military applications in detecting IEDs. Variants of the SSD include specific main route (i.e., roadway) clearance and land-mine EDDs. Some specializations are less well-recognized at this time, but becoming increasingly defined by the search task to be performed, such as cargo screening. Of course, many military working dogs (MWD) and law enforcement EDDs are dual-purpose or multi-purpose canines employed for multiple tasks including protection/apprehension or tracking, but these additional tasks are beyond the scope of this review; suffice to say that some specialty EDD tasks, such as the screening of persons, may be less compatible with dogs having the propensities necessary to perform these additional non-EDD tasks.

Specialization may be contextual in nature requiring that dogs exhibit characteristics particularly well-suited for working in particular conditions. This may be the case, for example, of EDDs for maritime operations working on and transferring between vessels, working in the confined spaces of those vessels, and in the loud environment of the engine rooms of those vessels. Some dogs that may not be behaviorally well-suited for particular tasks may possess very suitable characteristics for other tasks. For example, dogs unable to work in large crowds of people may be capable of specializing in interrogating cargo, where other characteristics, such as the ability to search vigilantly for long durations, has primacy. Some search tasks can also be delineated by the required concentration of explosive odor to be detected. For instance, for EDDs in the aviation security sector, trace levels of explosives are important to detect as compared to the SSD dog in a combat theater that may, in some circumstances, need to be conditioned to ignore trace levels of explosives. Such parameters may translate to the intrinsic propensity of a dog to engage in meticulous sniffing without which it may be difficult to condition it to detect trace levels of explosives or the PBIED EDD that may require an intrinsic propensity for air-scenting behavior as contrasted with ground- or object-scenting to perform the task successfully.

## Behavioral Characteristics

The behavioral characteristics required of EDD tasks can be generally broken down into three broad categories: detection characteristics, trainability/tractability, and environmental characteristics. Detection characteristics are those related to the style and intensity of interrogation and search for explosive odor. Trainability/tractability relate to the various cognitive, behavioral, and social characteristics necessary to be trained to perform the particular search requirements. Environmental characteristics refer to the collection of traits enabling a dog to work effectively in the particular search context, such as the high-stimulus settings of a large event venue, crowded mass transit stations, or military combat. In the following sections we aim to review these characteristics, as well as methods for evaluating the degree of expression of the various characteristics.

### Odor-Guided Behavior

Olfaction is undoubtedly a critical aspect of explosives detection. Olfaction is considered a primary sense for canines, but a large degree of variation in olfactory acuity exists due to differences in olfactory receptor genes and conformation based on selective breeding practices for morphological features designed to enhance olfactory ability. For example, differences in nose shape and population of odor receptor cells differ greatly between breeds selected for olfactory-based tasks and non-scenting breeds (9), leading to differences in olfactory threshold (12). However, sensory and morphological characteristics are considered secondary to behavioral characteristics in determining suitability as an operational detection dog (13). Rather, the specific type of odor-guided behavior used to identify and locate a scent, and a dog's propensity to use olfaction in general, is essential for effective operational search performance.

#### Search Technique

Different types of searching involve different search techniques, and thus the type of search technique desired will depend on the type of task. For example, air-scenting involves sampling odor molecules in the air, as opposed to on the ground or from objects, where the dog searches for the target odor by sampling the air currents in order to identify and work an odor to its source (9, 14). PBIED EDDs use air-scenting to detect airborne odor molecules, following the path of odor of the moving person. Due to the bilateralism of canine olfaction which allows dogs to determine the direction of an odor source by differential sniffing with each nostril, air-scenting is likely performed by detecting airborne scents in open areas without a scent trail to follow (9). Thus, an advantage of air-scenting is the ability to cover more ground in a shorter amount of time (15), and also allows dogs to locate some static targets more efficiently and directly by using air currents instead of following a path. There is a continuum, of course, between air-scenting and ground-/object-scenting as all odors are generally airborne; the difference is in the degree to which a dog attends to open air-space vs. its tendency to attend to and interrogate the ground or objects for target odor. Such tendencies are the result of both intrinsic qualities resulting from breeding and experience/explicit training. In selecting dogs for any detection task, the predominance of odor-guided behavior over other stimuli influencing behavior is a key foundational characteristic.

While all dogs are capable of air-scenting, which can be further fostered through training, breeds that naturally exhibit air-scenting (e.g., dogs selectively bred for upland game hunting) are often selected for such tasks. Potential advantages of using natural air-scenting breeds for detection work have not been systematically explored, and current evaluation methods do not typically account for these natural preferences (9). However, in a recent examination of the behavioral characteristics associated with dogs bred and trained for PBIED tasks, air-scenting ability assessed at 6 months was predictive of dogs' future placement as a PBIED EDD vs. traditional EDD, and was the only behavioral measure that distinguished the two types of outcomes at this age (16). This finding is consistent with the notion that air-scenting has a genetic basis, with such predispositions appearing earlier in development before extensive training may obscure differences. Thus, selecting dogs based on a natural propensity for the desired type of search method will likely result in reduced training time.

#### Propensity to Hunt

In addition to search technique, a dog's propensity for olfactory-based searching in general is an important characteristic in its success as an EDD. While most dogs can be trained to perform searches, as evidenced by the popularity of Nose Work^®^ as a sport for pet dogs of various breeds, some dogs are more naturally inclined to hunt with their nose without having to be trained to do so due to the intrinsically reinforcing nature of engaging in the hunt itself. Engaging in intrinsically reinforcing behavior in a non-functional context is thought to underpin the behavior of most working dogs, such as sled dogs racing as a form of play, or border collies showing of eye (17). A related but somewhat different example is that of the pointer, for which the stalk and pointing behavior has been greatly exaggerated and is presumed to be so strongly genetically controlled that external reinforcement is likely not necessary to maintain the behavior (18). Thus, the desire to hunt can be a powerful motivator for sustaining endurance and engagement during long searches where the probability of encountering a target odor, and thus receiving a reward, is low (3). Further, selecting dogs for which hunting is intrinsically reinforcing is likely to significantly reduce training time.

Even within hunting breed groups or within breeds, differences may exist as a function of the modern utility of the dog. For example, retrievers bred for hunting upland and non-waterfowl bird species (i.e., retrievers that also serve the function of a pointer and a flushing spaniel) hunt using their nose, detecting cryptic avian species (i.e., concealed or camouflaged). On the other hand, retrievers bred and trained for waterfowl hunting locate downed prey using visual cues and memory, relying less on their nose, in order to maximize the efficiency of retrieving the downed bird and minimize disturbance that may deter other birds in the area. This visual-based searching is even more enhanced in dogs bred and trained for competitions such as field trials and hunt tests where the primary focus is waterfowl hunting, which are further removed from the traditional utility of the breed, as is also seen in herding and sled competition trial dogs (17).

Evaluating a dogs' natural hunt ability can be measured by observing the pattern, efficiency, and intensity of the dogs' search. For example, when searching in a complex environment, efficiency will be improved if dogs ignore visual targets and only attend to odor cues in the air, using air currents to locate the source odor directly. In a test developed as a measure of search ability known as the Brownell-Marsolais scale (19), dogs are tested for their willingness to search for an object thrown into thick brush after varying intervals of time between the hiding of the object and when the dog is released to search. Higher scores are given for dogs that enthusiastically search the area without hesitation to enter the brush and locate the object. However, it is likely that other factors may influence performance on this test such as desire for the reward, memory for where the object was thrown, and sensitivity to the environmental aspects (i.e., entering and walking through the brush). In order to isolate natural hunt ability from other, likely interrelated, characteristics, attention should be paid to dogs' ability to hunt methodically using an instinctive pattern, efficiently searching areas with no encouragement or direction from the handler required. One could envision research that related wind current conditions, dog movement and sniffing, and ultimately, target detection that could enable the development of a standardized means of testing candidate EDDs for their relative efficiency and accuracy in using air currents to detect an explosive target.

### Reward Value

As discussed above, while some dogs find the opportunity to hunt reinforcing in itself, training a dog to perform a specific type of search task and to locate specific target odors typically involves using some type of reward (i.e., reinforcement) for performing the correct behavior. For example, teaching a dog to use a precise search pattern, respond to directional cues, detect an artificial chemical odor with no biological relevance, and communicate a find by performing a trained alert all require the use of operant conditioning to teach the desired behaviors. In order for something to function effectively as a reinforcer for a behavior (i.e., the behavior will be repeated in the future as a function of that reinforcer), the dog must regard the reinforcer as a high value reward- or at least more rewarding than competing sources of reinforcement available in the environment.

Food is a primary reinforcer for all organisms, meaning that an animal will work to obtain a primary reinforcer with no prior learning required due to its biological importance. Food rewards can be highly effective in training a dog, and is the preferred method used by the Bureau of Alcohol, Tobacco, Firearms, and Explosives (ATF) (20). However, the efficacy of food as a reinforcer can be influenced by individual differences in preferences and genetics, and may be impractical for use in some operational contexts. Furthermore, the reinforcing value of food decreases as satiety increases, though it can be increased through food deprivation. As such, training employing food rewards is often conducted prior to feeding using the meal ration during training, but satiety will eventually limit the number of repetitions that can be performed.

Toys are a popular reward used in detection dog training and are the preferred reward method of the military (20). The opportunity to chase, possess, and play with a toy is highly intrinsically reinforcing for some dogs. The reinforcing nature of playing with an inanimate object likely taps into the canine predatory motor sequence, a sequence of innate behaviors engaged in during the pursuit and apprehension of prey. Wolves engage in the full predatory sequence beginning with orienting toward the prey, triggered by its movement, and ending with dissection and consumption (21). Through selective breeding, the presence and intensity of parts of the sequence have been modified in domestic dogs, and in particular in working breeds which have and continue to experience strong selection for the expression of these patterns (21, 22). For example, dogs originally bred for assisting hunters (e.g., Spaniels and retrievers) exhibit exaggerated portions of the sequence related to chasing and grabbing, but not killing, as this part of the sequence would be counterproductive to the hunter. In herding breeds (e.g., border collies), the stalking and chasing portions of the predatory sequence are more greatly exaggerated (22). However, selective breeding has led to differences even within herders, such as German shepherds bred for protection (i.e., Shutzhund) which exhibit the orient, chase, and grab-bite (17).

For some breeds, such selection has led to engaging in these actions toward non-edible objects being reinforcing in itself, manifest as play behavior and an obsession-like desire for object-play (3). Moreover, the act of performing the behavior appears to be intrinsically rewarding and is unrelated to satisfying nutritional needs (23). The desire for object-play is then harnessed as a potent reinforcer allowing for the repetition of hundreds of trials without the risk of satiety (3), and functions as a powerful motivator to work over extended periods of time. In a comparison of three breed groups, retrievers were more likely to engage in solitary play with an object than livestock guarding dogs (which show no portion of the predatory sequence) and herders (22). Given such robust breed differences, object-play likely has a strong genetic basis and is likely to be evident early in development. Indeed, the tendency to retrieve an object has been shown to be predictive of future police dog suitability as early as 8 weeks of age (24). Similarly, a factor identified as “attitude toward predation” (comprised of willingness to chase, catch, and fetch a tennis ball as well as follow a dragged object) was predictive of police dog success as early as 7 weeks, and measures of fetching in 8-week old German shepherd puppies in a MWD program has been reported to be highly heritable (25).

The desire to maintain physical possession of an object is widely considered an important trait for a successful detection dog and likely reflects a high degree of intrinsic reward value (10, 11, 16). This characteristic, often termed “physical possession” or “object possession,” is often assessed by engaging the dog in tug-of-war play and measuring the dog's force and determination in maintaining its grip on the toy (26). However, when procuring dogs from gundog populations, in particular waterfowl dogs, it is important to differentiate natural possession (i.e., resulting from genetic selection) from a conditioned retrieve with a “soft-mouth” hold (i.e., resulting from negative reinforcement training). A dog with trained possession will typically only exhibit the beginning portions of the predatory sequence (i.e., retrieving and holding), which is less natural and likely to decay over time. A dog with a strong prey-related desire to possess the item will, if left to its own devices, engage in further behaviors such as thrashing and chewing.

A misconception in traditional working dog assessments is that a dog that relinquishes its reward after a target find has low reward value (i.e., lack of possession), but it may be that dogs with a high propensity to hunt, especially those that have been conditioned to perform multiple searches consecutively, may give up the reward for the opportunity to return to searching. It is also important to consider that such tests may be measuring multiple and potentially overlapping constructs; for example, a dog's engagement in a game of tug-of-war may be influenced by its desire to gain possession of the item, its desire to interact with the person playing, or both. Furthermore, it may be an inherent trait for dogs such as retrievers to have a propensity to return and drop a thrown toy at a handlers' feet in order to gain access to the opportunity to again retrieve the thrown object.

An analysis of tests used by the US Transportation Security Administration (TSA) for assessing dogs' suitability for explosives detection found that dogs' willingness to carry an object absent any external input reflected an underlying trait termed “independent possession,” which appeared to measure a different underlying construct than that termed “dominant possession,” the latter which was characterized by the duration and strength of grip during a game of tug. “Dominant possession” accurately predicted dogs' selection outcome whereas “independent possession” did not, indicating that the interactive nature of the tugging game may have reflected traits more important than independently possessing the object, such as a desire to interact with a person (26). Similarly, an analysis of the tests used to measure suitability of MWDs for the Swedish Armed Forces revealed that physical and social engagement were interrelated and both were predictive of training outcomes (27). Possession of an object and corresponding engagement with a handler during a tug game has also been shown to be predictive of suitability as an EDD as early as 6 months of age, indicating that this trait may be relatively genetically influenced and stable across development (16).

Some tests have differentiated between physical possession and “mental possession,” defined as the tendency to focus on an object or on the location where an object was hidden, and maintain focus over a period of time or despite distractions (6, 10, 26). This test is similar to the classic delayed-search task (28), and is likely a measure of sustained attention or memory. The more desirable the object is, the more motivated the dog will be to attend to its location over an extended period despite distractions or to remember the location of its placement after a delay. Thus, this test is probably influenced by a number of factors including reward value, attention, and arousal. Indeed, MacLean and Hare (29) found that performance on a delayed-search problem-solving task in which dogs were required to remember the location of a hidden reward after varying intervals was predictive of detection dog outcomes.

Another way to measure reward value is to assess a dog's persistence in attempting to obtain a reward. Persistence in dogs can be assessed by measuring the amount of time a dog spends attempting to gain access to an unattainable reward (e.g., a toy locked inside a container) before giving up, a test known as the “Unsolvable Task,” which has recently been used as a measure of detection dog suitability (29, 30). Persistence is generally considered a desirable trait as it likely reflects motivation for the reward, but in some cases, persistence can be a sign of learning difficulties related to an inability to flexibly respond to changing contingencies (31). For example, Dalal and Hall (32) found that greater persistence, measured as continued responding after reinforcement was discontinued (i.e., extinction), was associated with poorer olfactory discrimination learning. This suggests that high levels of persistence could be associated with an increased tendency to commit false alarms due to a decreased sensitivity to extinction. Further, Lazarowski et al. (30) found that glancing back and forth between the inaccessible reward and a nearby person during the unsolvable task as if requesting help was predictive of future placement as a detection dog, as opposed to dogs that persisted independently. Therefore, it may be that a balance between a strong desire to work for a reward with the ability to shift strategies when a response becomes ineffective is most desirable, which may be a sign of trainability (discussed below).

### Task Engagement

Many tests of working dog suitability include assessments of a dog's willingness and ability to stay engaged while performing a search (8). However, this general behavioral characteristic likely reflects a number of underlying traits rather than a unitary construct. For example, the desire to obtain the reward for completing the task as well as the reinforcing nature of the task itself is likely to influence individual willingness to work. Maejima et al. (33) found that drug detection dog success could be predicted by a general factor termed “Desire to Work” which consisted of several seemingly disparate underlying traits including increased general activity, ability to obey commands and concentrate during training, a greater degree of anxiety, and interest in a dummy object. Sinn et al. (6) identified a factor termed “Search Focus” which consisted of dogs' ability to search vigorously without handler input or interruption, using olfaction rather than vision, combined with physical stamina during the search (i.e., ability to search over large areas and long periods without physical signs of fatigue); despite the subtests reflecting a global construct related to search ability, the measure was not predictive of odor detection certification.

Task engagement may be best characterized as the dogs' level of independent engagement while searching. This is distinguished from propensity to hunt, which specifically refers to the dogs' willingness and ability for olfactory-based investigation, though a dog with a high propensity to hunt is likely to remain highly engaged in the task. However, task engagement also takes into account the level of the dogs' independence and ability to work without handler guidance or encouragement. A dog with low task engagement may require excessive handler direction in order to engage and remain engaged in the search, or may stay close to the handler and become easily distracted. A dog with high task engagement will remain engaged in the search independent of the handler until the target is located, immediately returning to continue searching after being rewarded.

Detection dog suitability tests also often measure distractibility, which is inherently part of a dogs' ability to stay engaged in a task. However, distractibility is thought to be a multifaceted construct and is not well-characterized in dogs (34). For example, one study found that in a population of drug detection dogs, aggression toward other dogs, low obedience, and a desire to play with humans were all related to a common construct thought to reflect distractibility (33), yet distractibility was not predictive of training success. Dogs may become distracted for a number of reasons, which may underlie different phenotypic traits. For example, disengaging during a search task could be due to impulsivity, or due to a general lack of interest (e.g., low hunt or reward value) (34). On one hand, whether a dog is able to be easily distracted from a task is important to know regardless of the cause of the distraction. However, the cause of the distraction may also be important. For example, a detection dog easily distracted by people may be able to work effectively in an environment without people, but knowing whether the distraction is due to a fear of people or an attraction of people would be important for accurate phenotypic characterization.

### Sociability

Detection dogs work as a team with a handler, and thus must be able to work effectively with humans. Responsiveness to human commands, e.g., influences the ease in which a dog is trained and is critical for the ability to be directed by the handler while working. For dogs that work down-range at a long distance from their handler, such as directionally-controlled Improvised Explosive Device Detector Dogs (IDD), the ability to respond to handler commands is imperative to the team's success and safety. Indeed, a recent study found that the ability to utilize human gestures in a problem-solving task was associated with desirable IDD outcomes (29). For these reasons, breeds originally selected for working cooperatively with humans (e.g., herding dogs, gundogs), selected for their ability to work while maintaining visual contact with their human partner and taking commands from a distance (35), tend to be favored for a variety working roles involving working as a team with a person. Conversely, “independent worker breeds” (e.g., scent hounds, livestock guarding dogs) were bred for working independently with minimal human interaction. For example, bloodhounds were selected for a steadfast persistence in pursuing an odor trail independently and over long distances, making them excellent tracking dogs. However, the trade-off is that this “single-mindedness” which allows them to focus entirely on the odor and ignore distractions can make them stubborn, disobedient, and difficult to train (3). For this reason, scent hounds are rarely used in explosives detection despite their purported superior olfactory acuity.

The ability to cooperate also relates to a willingness to please. Wilsson and Sundgren (36) described this trait as “the tendency to be influenced by the handler without being given a direct command or sign,” and found breed-related differences in scores for this trait. Scores were higher in Labrador retrievers compared to German shepherds, and was the most heritable trait for the labs (37). Cooperability/willingness to please was found to be a separate behavioral trait than a willingness to make contact with people, termed affability, which was also found to be higher in Labradors than German shepherds. The authors attributed these breed differences to the genetic history of the Labrador, originally used as hunting dogs that worked closely with their human partners, while German shepherds were used for herding and livestock guarding and more recently as police and protection dogs. Despite the importance of dogs' desirability to interact and work with people in their effectiveness as a team, EDDs should not be so attracted to people to the point that they become distracted (11). While play with the handler is likely to be reinforcing and highly effective for training, the task itself must be more rewarding in order for dogs to work effectively.

Similarly, dependence on the handler is an undesirable characteristic as detection dogs need to be able to work independently without constant guidance (11). Dogs are incredibly sensitive to human body language and other social cues, and so too much attention to the handler could interfere with the dogs' ability to make independent decisions (38). In a recent study, adolescent candidate explosives detection dogs that ignored a human's inaccurate pointing gesture that conflicted with olfactory information were more likely to be selected as EDDs in the future than dogs that followed the deceptive gesture (39). On the other hand, dogs that are too independent may be stubborn and difficult to train or control. For example, as mentioned above, candidate EDDs that independently persisted longer on an unsolvable task (attempting to obtain a reward from a locked container) were less likely to be selected for working roles in the future than those that looked to the handler, considered a sign of soliciting help, suggesting that some degree of social sensitivity to people is important (30). The degree of desired independence likely depends on the type of task, where dogs trained to work off-leash need to be able to range far ahead of the handler but be responsive to directional controls given by the handler at a distance.

### Trainability

The speed and ease in which a dog learns a new behavior or task, or trainability, is clearly an important characteristic as rapid learning will lead to faster and thus more efficient training. A greater desire for the reward will enhance attention to the task, motivation to remain engaged, and thus increase training efficiency (9). Thus, trainability is likely a multifaceted construct involving several other processes like attention, as learning also requires sensitivity to changing contingencies. Trainability may therefore be a difficult construct to measure using a single test. Trainability in pet dogs as assessed by The Canine Behavioral Assessment and Research Questionnaire (C-BARQ), a widely used assessment of dog behavior with established reliability and validity, is defined as an aggregate score including attention to the owner, obedience to simple commands, fetching objects, responding positively to correction, and ignoring distractions, and has demonstrated high heritability (40). Trainability measured by the CBARQ has also been shown to predict MWD success rate, and correlated with a behavioral test of “physical engagement” which consisted of tug of war, chasing, interest in object, and persistence in searching for a hidden tennis ball (41). Trainability is likely a combination of the various traits described thus far, which, when evaluated in conjunction, provide a strong predictor of working dog success.

### Emotional Reactivity

#### Arousal

Detection dogs are typically selected for high activity levels, and high energy is thought to result in strong motivation and willingness to work. Handlers of actively working police dogs in the UK reported higher levels of “energy and interest” observed in their dogs compared to reports of those withdrawn from service for behavioral reasons as well as a population of pet dogs (34), suggesting that higher energy levels may be associated with desirable working dog characteristics. However, high energy and activity is often associated with increased arousal. For example, Belgian Malinois bred as MWDs exhibit high levels of excitement often resulting in spinning behavior when kenneled, which has been reported to be higher in individuals with better work performance due to a stronger desire to work (42). Maejima et al. (33) found that higher levels of anxiety were associated with a stronger desire for work and successful certification as a drug detection dog. Given that physiological arousal is closely associated with stress (e.g., increased cortisol) (43), it may be that selecting for high levels of energy and arousal carries with it an increased general reactivity.

While arousal can reflect either negative (e.g., stress) or positive (e.g., excitement) emotional states, high levels of arousal regardless of the underlying affective mechanism can interfere with the ability to perform a task. The phenomenon known as the Yerkes-Dodson law (44) has been well-established in humans, and recently in dogs (45), demonstrating that there is an optimal level of arousal for successful performance on a task where increasing arousal can improve performance on a task up to a certain point, after which performance begins to decline. This effect varies by individual baseline arousal level; for example, the inhibitory control abilities of service dogs with low baseline levels of arousal benefited from a boost in arousal, whereas, the performance of pet dogs with higher baseline arousal suffered when arousal was increased (by exciting the dog) (45). Further, increased arousal can impair learning, memory, and decision making (43). Increased arousal is characterized by activation of physiological responses, such as increased breathing and heart rate. Thus, increasing arousal may result in heavy panting, which reduces olfactory ability as dogs are not able to sniff and pant at the same time (43, 46). While high energy levels are important for sustaining motivation during long searches, excessive arousal may interfere with endurance. For dogs with high baseline levels of arousal, the excitement of searching and being rewarded with play could increase arousal to suboptimal levels and interfere with stamina. Therefore, on-task arousal should be evaluated while working over a period of time, assessing cumulative effects. Adverse signs of arousal that may interfere with performance include open-mouth searching, whining, salivating, frantic searching, agitation, decreased ability to safely navigate the search area, and difficulty handling, increasing over the course of the task. Importantly, low arousal can also be suboptimal if dogs are uninterested or unmotivated, and thus arousal should be evaluated in conjunction with other measures like task engagement.

The anticipation of beginning a task can also lead to an increase in arousal, which can interfere with subsequent performance. For example, if a team must wait before entering a building to start a search, the anticipation during the wait can be stressful for some dogs. Anticipatory arousal can be measured using the same tests traditionally used to measure dogs' ability to locate a thrown object after a delay, but by measuring the dogs' behavior during the delay while restrained such as whining, barking, spinning, aggressing toward the handler, and other behavioral signs of stress indicating the dogs' inability to manage the frustration of anticipation. For example, how much time a dog spent running and restless while restrained by the handler was indicative of “energy management,” considered an important trait in selecting dogs for explosives detection (26).

A dogs' ability to manage arousal levels while off-duty, referred to as “off-duty calmness,” has been reported by handlers as an important but often overlooked characteristic for EDDs (10). This may be due, in part, to a larger systemic problem related to a lack of feedback to procurement teams from trainers and operationally deployed teams. For dogs living in homes with their handlers, the ability to adapt to the home environment and remain calm while off-duty is clearly important to the dogs' ability to adapt to the handler's home life and ease of management. For dogs living in kennels, the ability to relax when not working may be indicative of effective energy management or general anxiety. One study examined the behavior of guide dog candidates in the kennel and found that a greater amount of time spent resting was predictive of certification (47). The authors speculated that resting during the evening allows for better concentration during training the following day. Further, as in humans, sleep has been shown to be important for memory consolidation and learning in dogs (48, 49). Unpublished data indicated a similar pattern in a population of EDDs, in which dogs that had been successfully selected for service spent a greater proportion of time resting in the kennel than dogs that had been rejected, and that the amount of time spent moving in the kennel was associated with poorer reactions to novel objects, visual startles, and people during a behavioral test (50). In this case, a lack of resting in the kennel likely reflects underlying anxiety and hypervigilance that interferes with the ability to relax. Thus, selecting dogs that are able to “turn off” and appropriately channel arousal is critical.

#### Fearfulness

Detection dogs are exposed to a range of unpredictable stimuli in the environments in which they work. Therefore, an aspect of behavior that is critically important to their success is resilience toward potential stressors, referred to by a number of terms including environmental soundness (16), environmental sureness (27), environmental stability (26), nerve strength (19), emotional reactivity (51), courage (36), and sensitivity to aversives (8), and are commonly reported as primary reasons for rejection or disqualification across working dog programs (16, 52, 53). Fearfulness can be detrimental for most working dogs, but especially for EDDs working in mass-transit areas with large crowds of people, noisy ambiance, or urban environments with a large variety of novel stimuli to be encountered. For this reason, the level of soundness required is likely comparable to that of guide dogs; however, guide dogs must be wary of potential dangers in order to safely navigate their handler and such wariness has been shown to be predictive of future guide dog selection (54), whereas EDDs do not have this level of responsibility.

Evaluating environmental soundness typically involves presenting dogs with a series of anxiety-provoking situations, with the goal of identifying behaviors during the tests that may reflect the dogs' ability to work effectively in a range of environments (8). Fearfulness in dogs is often characterized by *approach-withdrawal* tendencies, including avoidance of a stimulus, exploratory behavior, and reactions toward stimuli (8, 27, 47, 51, 55–57). Other measures include identifying the presence and severity of specific behavioral indicators of fear (e.g., posture, tail position, lip licking, freezing) in response to stimuli as a measure of *sensitivity to aversives* (8). Both initial reaction and subsequent recovery may yield important information, as the initial startle response likely reflects general autonomic nervous system sensitivity, whereas recovery reflects the ability to cope with the stressor (8). Repeating an exposure can also be informative, as decreased sensitivity upon repeat exposures may be indicative of adaptability and desensitization, and increased sensitivity indicates an inability to cope. For example, Tomkins et al. (47) found that the longer it took a dog to settle after the third repetition of an acoustic startle (a metal plate hitting a concrete floor), the less likely it was to succeed as a guide dog. This suggests that a transient sign of fear with immediate recovery may be acceptable and indicative of resilience (58). The degree of acceptable fearful behavior likely depends on the nature of the dogs' role, or the goals of the selection. For example, greater fear responses may be acceptable for a dog that will be deployed in lower-intensity situations and if the specific fear is likely amenable to overcoming with training. If selecting for breeding, a lower degree of fearfulness will be more important as fearful behavior is known to be heritable (53). It is also important to measure behavior using a wide range of tests rather than a single component, as fearful behavior in isolated incidents may be less problematic than consistent fearfulness across a range of contexts which could be indicative of a more stable fearfulness trait (59). Further, aggregate scores of canine behavior assessments have been shown to be more predictive of future behavior than single measures (24, 54, 60). Indeed, fearfulness has been characterized as a Fear/Reactivity personality dimension in dogs (61), and influences a range of important working dog outcomes (52). For example, Svartberg (57) found that a personality dimension of shyness-boldness reflected fearfulness as well as general learning ability, indicating that boldness (i.e., more exploratory and outgoing) likely facilitates learning due to encouraging interaction with the environment, persisting against challenges, and being less distracted or inhibited.

##### Social fears

Studies have indicated the presence of two separate and distinct aspects of fearfulness, one relating to social fears (e.g., toward unfamiliar humans and dogs) and another to non-social fears (e.g., inanimate objects) (52, 62, 63). For EDDs that will be expected to work in environments where they may encounter people or other animals, such as in airports, public venues, and mass transit areas, social fears may interfere with the ability to work effectively. For dogs that work in close contact with people, such as passenger screening dogs, friendliness toward people may be an important consideration regarding public perception and level of comfort (11).

A common way to measure social fear toward people is to evaluate dogs' greeting behavior toward an unfamiliar person, such as willingness to approach a stranger, as well as body posture and other fear behaviors during the approach or during interaction with the stranger. Other stimuli have been used when the safety of the human is a concern, such as human-like dolls or dummies (64). Fearfulness toward other dogs has been measured by evaluating the dogs response to another “stimulus dog,” a fake model dog, a picture of a dog, or a mirror reflection, though any artificial representation of a dog will not provide social information (e.g., odor, movement) that could influence responses (64). Social fears have been shown to elicit a greater fear response than inanimate objects in dogs, with dogs only vocalizing in response to social fears suggesting a communicative intent of the behavior (65). It can then be speculated that any vocalizations toward inanimate objects may be due to the dog perceiving it as a person or animal.

##### Non-social fears

Detection dogs also encounter a variety of non-social stimuli in their working environments that could potentially interfere with their ability to complete task. Non-social stimuli are typically characterized as mobile/animated, immobile/inanimate, acoustic, and visual (64) and reflect a separate category of fear than social fears (63). Below are various types of non-social fears relevant for EDDs and common ways to test for them.

*Tactile* Detection dogs must be able to continue searching without hesitation across a variation of surface textures (19). For example, EDDs completing a building search must be comfortable walking across varying surfaces such as slick flooring, and searches in urban areas may require traversing open grates or unstable footing. In addition to underfootings, detection dogs must also not show sensitivity to body contact, which could inhibit the ability to search in tight spaces, navigate over an obstacle, or under objects hanging overhead. In this regard, an explosives detection dog's confidence encountering a variety of tactile stimuli is similar to that required by search and rescue dogs required to navigate over rubble piles and unstable structures.

*Elevation* Explosives detection dogs must be able to climb tall structures and search on elevated surfaces if necessary. While a fear of heights in most mammals represents an innate and evolutionary-based aspect of self-preservation (59), fear that prevents a detection dog from searching an area of concern can be a performance-limiting factor. Fear of elevation can be assessed by observing dogs' willingness to approach a ledge or to jump off of a raised surface (e.g., out of a truck), or more formally using a catwalk or elevated maze (59). For example, King et al. (59) found that dogs spent significantly more time in the closed arms of an elevated (1.5 m high) plus maze than the open arms, suggesting that the open arms were somewhat aversive.

*Stairs* Behavioral tests for working dogs also typically involve testing dogs' willingness to ascend and descend stairs, which may or may not reflect fear of elevation. A variety of types of stairs should be tested including open-backed or open-grate stairs, which may invoke a greater elevation-based response than closed stairs. Approach of the stairs as well as behavior while on the stairs should be assessed as the ability to traverse the stairs in itself may not be indicative of a lack of fear; for example, a dog fearful of stairs may rush over them. Fear of stairs has been shown to be unrelated to other non-social fears, suggesting that a fear of stairs may represent a different underlying fear that develops separately from other fears that appear to be related (e.g., unusual or unfamiliar noises or objects) (63). This would suggest that if fear of stairs is not related to an underlying fearfulness trait, that with sufficient experience and training on a variety of types of stairs the fear can be diminished. However, in contrast to this, Wilsson and Sinn (27) found that fearful behavior on a metal staircase was associated with fearful behavior in a dark room as well as in response to an acoustic startle test, all of which when combined appeared to reflect a trait considered to measure “environmental sureness.”

*Auditory* Noise sensitivity is a common reason for release from working dog programs (66). For detection dogs, such sensitivity can be detrimental to the ability to work effectively as noises from machinery, traffic, blasts, gunfire, and general urban noises (e.g., loudspeakers, people talking) can be distracting or produce anxiety. A popular test used widely by working dog programs is the acoustic startle test, in which dogs' response to a sudden and loud noise is measured. The acoustic startle response is a fast contraction of the muscles elicited by a sudden and intense sound and is present across all mammals (67). Common tests of acoustic startle in working dogs include response to a gunshot, metal objects being dropped on hard surfaces, or other sound blasts (24, 55). Fearful reactions to noises such as fireworks and thunderstorms are typically evidenced by freezing behavior (68), while the latency to recover from an acoustic startle may be a reliable predictor of future success (47, 69). Reduced fearfulness and greater exploration in response to noise at 7 weeks of age was predictive of success as a police dog as an adult (25). Another study found that responses in a gunshot test were not predictive of future police dog outcome, which was attributed to likely prior desensitization because all puppies tested had been exposed to gunfire as part of their socialization (24).

While acoustic startle can be greatly diminished through proper desensitization during early development (70), evidence suggests that this response has a genetic basis. For example, (37) found breed differences in working dogs' responses to frightening situations and gunfire in which Labrador retrievers scored higher (less reactive) than German shepherds. Again, this difference was attributed to the breed history of the retriever, selected for working closely with hunters and withstanding gunfire at close range. Indeed, breeds commonly used for sport-hunting (e.g., Labrador retrievers, Cocker Spaniels, Springer Spaniels) have a reduced tendency to show an acoustic startle response. Researchers have speculated that genetic variations associated with hearing loss may be responsible for reduced startle responses in hunting breeds, but confounds of exposure resulting in habituation or possible damage to the auditory system cannot be ruled out (71). Thus, it is possible that selection for dogs that are less responsive to gunfire has actually modified physiological or anatomical characteristics making some sub-populations of dogs less sensitive to loud sudden noises.

*Visual* Similar to acoustic startles, visual startles in which an object suddenly appears are commonly used in tests of working dog suitability, such as an umbrella opening, a dummy popping up, or a bag falling in front of the dog. The severity of the startle response, time to recover, and exploration of the object are then measured. A visual startle test measuring the dogs' reaction to a person suddenly jumping out in front of the dog was found to be predictive of future police dog performance as young as 12 weeks of age (24). Reactions including running away and avoidance were associated with poorer outcomes whereas not attempting to run away, and even barking or trying to attack the stranger, was associated with successful outcomes. The type of reaction is clearly related to the nature of the role, where in dogs with a protection role confronting a potential threat aggressively is more desirable. Similarly, Foyer et al. (58) found that stronger emotional reactions and higher levels of cortisol in response to potentially fear-inducing stimuli was predictive of placement as an MWD. For explosives detection dogs, an unremarkable reaction would be most desirable. In particular, for EDDs working around crowds of people, this type of reaction would be undesirable and potentially dangerous. Further, behavior that may be perceived as overly confident, such as moving toward a threat, may actually reflect the dog attempting to actively control the situation driven by fear as fearfulness is sometimes exhibited as an active reaction or agitation (54, 55).

*Novelty* Because EDDs will likely encounter novel situations on a daily basis, behavior in unfamiliar environments or toward odd or unfamiliar objects such as statues, animated objects (e.g., race car), and large or oddly-shaped items (e.g., beach ball, umbrella, rocking horse) are commonly used to assess working suitability (53). Novel object tests differ from visual startle tests in that they are not intended to elicit a startle response, but rather measure the dogs' willingness to approach an ambiguous object. Indeed, King et al. (59) found that responses to novelty and responses to startles appeared to measure two different types of fearful behavior. When encountering an object that is novel, a dog must make an appraisal as to whether the object is benign or potentially dangerous. In this sense, novel object tests may be similar to the cognitive bias test which assesses animals' responses toward ambiguity and serves as a measure of positive or negative expectancy (72). In this task, approaching an ambiguous stimulus in the same manner as a stimulus with a positive association (i.e., previously rewarded) is indicative of a positive expectancy, whereas approaching in a manner similar to a stimulus with a negative association (i.e., previously unrewarded or punished) is indicative of a negative expectancy. In dogs, negative cognitive bias is associated with negative emotional states (73). Thus, how a dog approaches a novel object may be indicative of its bias toward expecting positive or negative outcomes.

It is likely that the appraisal of the object as a threat or not involves perception of the object as a predator. Thus, novel object tests that use animal statues or objects with facial features (e.g., large eyes) likely tap into predator avoidance responses. Avoidance or defensiveness toward novelty is a well-established fear response in many animals and is of clear adaptive value, and novel objects with intense characteristics, movement, and unpredictability are likely to elicit predator-related fear responses (59). While predator-related fear is considered an innate and adaptive response across mammals, it must necessarily be reduced in detection dogs that will be expected to work effectively regardless of what is encountered. In this sense, the increased behavioral requirements for EDDs may result in selecting dogs with reduced self-preservation behavior; the consequences of which may need to be taken into consideration for the safe operation of an EDD.

### Puppy Tests

The ability to test a puppy in order to reliably predict future behavior has been called a “holy grail' of dog research (13). For working dog programs, the ability to make decisions about breeding, training, or career paths as early as possible would significantly reduce the amount of time and costs involved. Unfortunately, research on the reliability of puppy testing is mixed, with many studies failing to find any consistent relationships between puppy and adult behavior [56, (37)]. In general, adult behavior is difficult to predict in puppyhood due to the continued interactions between neurological, environmental, and genetic influences across development (56). However, a few studies have found that certain aspects of behavior can be predicted in puppyhood (25, 54, 60). Behaviors exhibited during puppy tests that hold predictive validity are likely to be strongly genetically based, as such stability would indicate strong resistance to change due to environmental influence and maturation (13). For example, fearfulness is considered a core personality trait in dogs that can be identified early in working dogs and is relatively stable across development (52).

In general, the predictive power of puppy tests increase with age (53) and may be able to more accurately predict behavior when combined with other measures compared to a single measure (60). Aspects of the early environment such as proper socialization have been shown to be strongly associated with adult behavior (74, 75). Thus, rather than testing puppies for specific reactions, it may be more informative to know what kind of experiences and exposure the puppy received during early development. Further, tracking a puppy's behavior across development rather than testing at one time may be valuable. For example, McGarrity et al. (26) found that a construct that combined multiple measures including the ability to focus on a location where an object had been hidden, carrying a toy without handler engagement, grip of a toy during a game of tug, and performance during a search task was predictive of selection as an EDD, but only when assessed over time. Specifically, the overall score was not predictive but rather an increase in the score across the first year of life was, suggesting that tracking improvements in performance across development may be more informative than focusing on a single time point (26). Routinely evaluating behavior may also be useful for identifying deficiencies in order to develop targeted training interventions and to monitor progress in response to changes in training and breeding practices (56).

### Types of Assessments

#### Behavioral Assessments

The majority of working dog assessments, and the majority of those reviewed above, utilize traditional behavioral assessments consisting of a series of sub-tests aimed to measures aspects of temperament. However, a lack of consensus and standardization in regard to terminology, test quality, stimuli used, and variables measured makes the ability to make meaningful comparisons across groups and extracting results challenging (64).

Behavioral tests are often scored using subjective ratings methods in which an observer makes a judgement of global behavior on a Likert-type scale to indicate the degree of a behavior, e.g., in terms of its frequency, desirability, or strength (26). Alternatively, coding methods involve quantifying specific instances of behaviors that occur during the test (e.g., barking, cowering, jumping) which may be more objective than rating methods (26). While some studies have found consistencies across rating and coding methods, the optimal method may depend on the situation (26, 27).

#### Questionnaires

A number of questionnaires and handler surveys have been developed as alternatives or adjuncts to behavioral testing, which is time consuming, less cost effective, and may not accurately capture behavior that occurs outside of the test situation (56). Handler reports of dog behavior using the C-BARQ has been successfully used to predict working dog suitability (41) and guide dog training success (56, 76, 77). The Positive and Negative Affect Scale (PANAS), developed as a way to measure sensitivity to rewards and punishers, and the Dog Impulsivity Assessment Scale (DIAS) (78), assessing factors related to impulsivity in dogs such as behavioral regulation, aggression, and responsiveness, have both been successfully used to identify important characteristics in police dogs (34). An advantage of a survey such as this is that data can be collected without having to expose dogs to potentially stressful situations, which could subsequently affect future behavior (79). A disadvantage of surveys is that accurate reporting requires sufficient knowledge of the dog (e.g., its trainer or handler), which is not always feasible when assessing large numbers of previously unseen dogs in a mass procurement activity, in which even the vendor of those dogs may not have prior experience with those dogs, and also requires honesty and accuracy by the person reporting (8). Though survey reports may not be the most feasible selection tool alone, they may serve as a valuable measure for validating existing behavioral tests.

#### Cognitive Measures

Recently, researchers have applied measures of cognitive ability (i.e., problem-solving and information processing) to the assessment of working dog suitability. Many aspects of cognition are likely involved in working dog tasks including memory, behavioral flexibility, mental representation, self-control, and communication, and therefore may represent quantifiable and objective metrics for evaluating individual differences in detection dog success (29, 80). For example, as discussed above, measures of socio-cognitive abilities have indicated that social communicative behaviors are related to detection dog trainability (29, 30, 39). Other studies have found that measures of non-social cognition are predictive of working dog performance, such as inhibitory control, problem-solving, and short-term memory (29, 66, 81, 82). Thus, assessments of cognitive abilities identified as contributing to working dog success show promise as valuable complementary measures to traditional evaluations for improving the selection process (29).

## Conclusions

**Figure 1:**
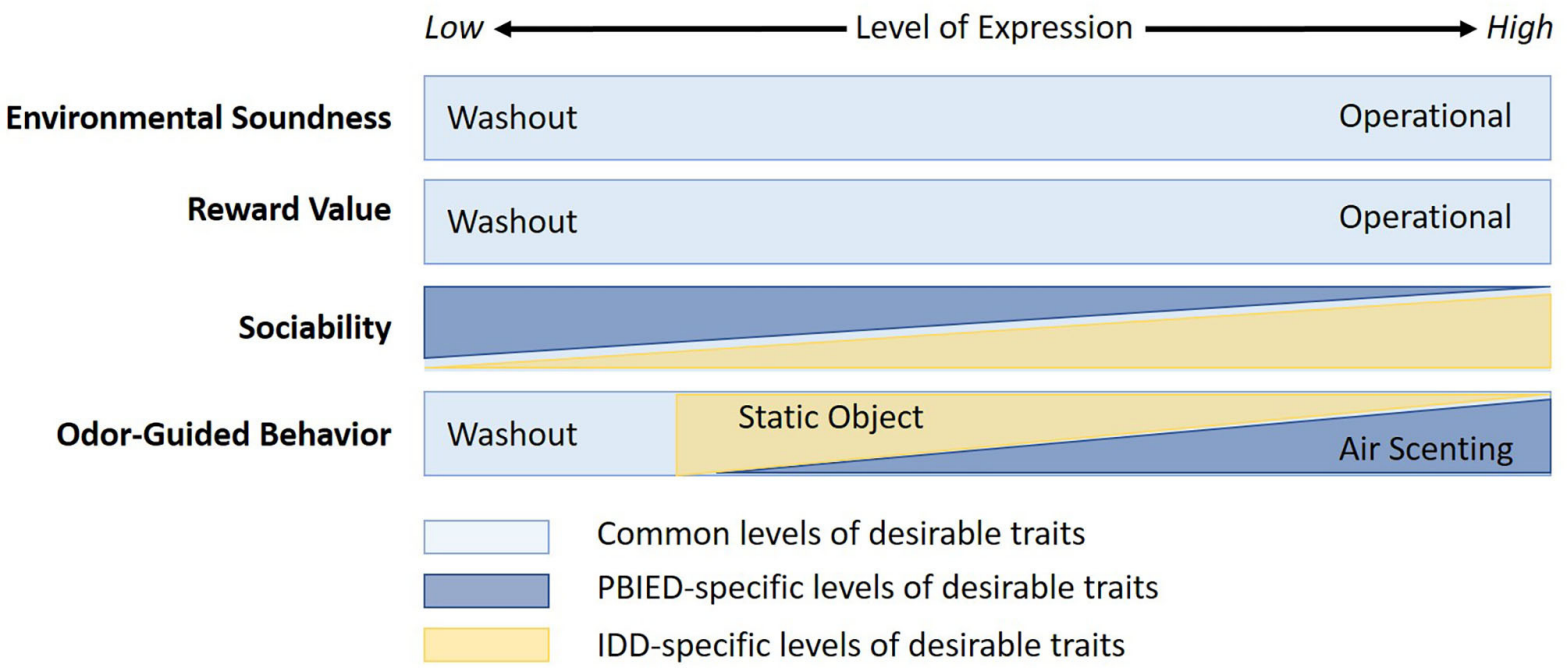
Theoretical representation of the critical behavioral characteristics and optimal levels of expression necessary for explosives detection dogs, where Operational refers to dogs deployed in the field and Washout refers to dogs unsuitable for such roles.

In this review, we highlight the behavioral characteristics that appear to be critical in the selection of EDDs. Many of these characteristics are similar to those desired in other types of working dogs such as search and rescue, conservation, protection/patrol, and even assistance and guide dogs, which also require high levels of motivation, trainability, and the ability to work in potentially stressful environments. However, we suggest that there is an ideal constellation of characteristics for EDDs that is somewhat unique, which is likely also the case for other types of working dogs. The constellation of desirable characteristics for explosive detection is defined by the degree and suitable balance of the expression of particular characteristics, and the optimal balance of this expression will vary based on the specialized explosive detection application (Figure 1). Specialization is rapidly becoming more normative than the general or “standard” EDD, which is indicative of technological advancement of explosive detection dog capabilities.

The behavioral characteristics examined in this review may be divided into three broad categories, detection characteristics, trainability, and environmental soundness. Detection-related characteristics include odor-guided behavior such as the innate propensity for hunting and the type of search technique. Trainability comprises multiple, likely overlapping traits that will influence a dogs' ability to learn, such as reward value and sociability. Environmental-related characteristics include emotional reactivity subcategories of arousal and fearfulness. These are likely not unitary categories and there are probably significant interactions within and between performance and environmental characteristics.

Explosive odors do not have any particular biological relevance to dogs and thus are not of any intrinsic interest for them to detect. Therefore, it is imperative that candidate explosives detection dogs exhibit a strong combination of odor-guided behavior and very high reward value in order to build the contingency between detecting explosive odors and obtaining a desired reward. Although food is inherently reinforcing and is used by some organizations for reinforcing explosive detection performance, managing satiety and diet complicates its use and the opportunity to play with a toy and/or the handler is the predominant reinforcer used in operating explosives detection dogs. Furthermore, it would appear that dogs for which delivery of toys is a highly effective reinforcer have concomitant characteristics, such as a propensity to hunt with their nose, important to detection dog performance. The degree of possession of a toy appears to be a useful metric of the potential of a dog for being successfully trained and employed as an EDD, but the nature of such possession (e.g., “independent” vs. “dominant”) may reflect multiple traits that have differential predictive value. Once the contingency between detecting explosives odors and obtaining a desired reward is established, it is desirable in EDDs that such conditioning makes the opportunity to engage in searching a preferred activity.

A dog's willingness to stay engaged or focused on searching and vigilant for alerting to target odors in the midst of distracting stimuli is also an important metric in assessing its potential as an EDD. Resilience in searching in the absence of handler encouragement and prompts to work, which can lead to a dependence on handler cues that can increase false alerts, is a particularly important characteristic for explosive detection performance because of the relatively low rate of encountering targets.

It is important in EDDs that independent task engagement and lack of distractibility does not come at the expense of trainability. For example, although it is essential that PBIED detection dogs remain engaged in searching around people, it would appear that social sensitivity is an important characteristic for following handler directions (39) and being successfully trained as an EDD. Thus, a balance of underlying traits, such as social sensitivity and co-operability, which seem to contribute to trainability, with that of independent task engagement, is needed for optimal EDD performance. Though the focus of this review is on dog characteristics, it is important to consider the impact the human side of the dog-human dyad can have on the performance and success of a detection dog. For example, search errors are often due to handler error, such as handler-induced false alerts (38, 83) or missed targets due to the handler interfering with the dogs' ability to adequately search an area (84). Handler stress can also influence dogs' search performance (85, 86), and working with an unfamiliar handler can be stressful for dogs leading to reduced search accuracy (87). Other characteristics related to the experience and skill of the handler, such as timing of reinforcement delivery, consistency in their interaction with the dog, and training methods will also be critical to the efficiency of training and ultimately the success of the dog, though research in this area is lacking (88). Future studies are needed to identify the attributed of effective handlers and optimal methods for their selection and education.

Equally important and arguably of greater difficulty to define and assess than the performance-related characteristics summarized above is emotional reactivity, often classified as “environmental” characteristics. The difficulty in defining and assessing emotional reactivity in relation to the potential of a dog to perform explosive detection is in large part due to high levels of energy or activity associated with arousal being both a positive, necessary, attribute to performing detection work and such levels of arousal carrying along with it increased generalized reactivity to stimuli that interfere with performance. High levels of general arousal may also interfere with learning and performing a task. Low levels of general arousal are associated with low levels of activity and motivation to perform a task. Thus, it is not a balance of high and low arousal that appears to be ideal but rather arousal organized about and directed toward engaging in searching and detecting odor targets. Anticipatory pre-work and off-duty high arousal detract from performance as does arousal associated with reactivity to stimuli that may manifest as fearfulness of both social (e.g., negative – fearful reactivity to novel people) and non-social (e.g., negative – fearful reactivity to loud sudden noises) stimuli. Such fearfulness is decidedly incompatible with performing explosive detection in the most frequent context for such work, the modern urban terrain.

In our experience, evaluating environmental soundness in highly motivated dogs is further complicated by such dogs often lacking awareness to stimuli in the environment when they are engaged in searching. This effect may be the result of inattentional blindness, which refers to the failure to notice unexpected stimuli when engaged in a task demanding high levels of attention (89). Although inattentional blindness has not yet been explored in dogs, a recent study found that horses trained to expect a reward in a particular location show a reduced startle response to a novel stimulus compared to those that were not given such experience (90). Therefore, we suggest it more useful to examine emotional reactivity while dogs are not and have not immediately been engaged in searching, and to conduct such evaluations in an area not associated with expectation of reward. This is more difficult than it may first seem because, for dogs engaged in training, the context of transportation, location, and presence of trainers/handlers all tend to predict the opportunity to engage in searching, find target odor, and obtain their reward. Although environmental interest outside the context of searching may at first seem to *not* be critical, in operational deployment there is considerable down-time, which becomes readily discernable to the dog, between searches in operational venues when emotional reactivity to stimuli may occur that sensitizes the dog to those stimuli such that it interferes with performance. Future research is needed to examine such contextual factors that may influence performance during behavioral assessments as well as the effectiveness of environmental socialization.

In selectively breeding dogs for detection, performance characteristics appear to be more readily enhanced by selective breeding than environmental characteristics, with lack of environmental soundness being the predominant reason for failure of dogs to succeed in explosives detection (16). Anecdotal reports in our contact with the explosive detection dog industry suggest that, particularly at the extreme of the performance continuum such as PBIED and SSD (i.e., off-lead IED detection) specializations, the normative reason for failure are emotional reactivity issues. Although this may be the result of differences between performance and environmental characteristics' sensitivity to selective pressure, we posit that it is much more likely the result of the difficultly in defining and disentangling positive and negative aspects of arousal with current assessment techniques.

Understanding how particular behavioral characteristics are related to explosive detection performance is key to a technology for assessing dogs for such service. The foundation of such an assessment technology will be the extension of the important research efforts examined in this review evaluating this relationship. Unlocking the potential of such assessments will further depend on a deeper look into the phenomenology of the characteristics themselves in order to better define, disentangle in several cases, and measure those characteristics. Development of a standardized and accurate assessment technology that can be applied across programs will be critical to increasing the supply of suitable detection dogs and improving detection technology overall.

Puppy assessments predictive of potential explosive detection performance would allow for significant efficiencies in managing resources and optimizing the value of every dog bred for working purposes by directing it toward a successful career path. Research suggests that some characteristics, such as fearfulness, observed in puppies are relatively stable across time suggesting strong genetic determinants (52). Characteristics that are emergent and likely significantly influenced by experience may be better assessed by multiple observations across time and trends, such as stability or improvement, and may be more predictive than any single time-point observation. Our review suggests that there is a useful historical base and some momentum for more targeted research in early assessment activities that promises to advance early prediction of the potential of dogs for explosive detection tasks.

Current procurement of candidate dogs for explosive detection usually utilizes behavioral assessments based on traditional conceptualizations of working dog characteristics for which subjective ratings are assigned. Different organizations use different assessments, which in part logically reflects the parameters of the particular organization's explosive detection mission. However, there is also considerable variability in terminology, testing techniques, and subjective ratings making it difficult to make meaningful comparisons across assessments.

Validated questionnaires such as the C-BARQ and PANAS have been shown to predict, among other behavioral outcomes, working dog suitability. Questionnaires are unlikely, however, to replace direct immediate behavioral assessments for selection of candidate EDDs because they require that the respondent has historical knowledge of the dog's behavior and the impracticality of such a respondent's judgement being impartial or being perceived as such to the receiving/procuring party. Nonetheless, elements of these validated questionnaires in combination with direct observation of behavior might be combined to potentially enhance the predictive value of assessments for selecting candidate explosive detection dogs.

Finally, recent research suggests that the incorporation of measures of problem solving, information processing, memory, and inhibitory control adapted from the cognitive sciences hold significant promise of providing more objective and quantifiable metrics indicative of suitability for explosive detection tasks. Another potential advantage of cognitive measures is that they may access underlying, immutable characteristics related to performance.

There is a need of a selection technology to advance the production and utilization of explosive detection dogs. Our review suggests that this technology is dependent upon ongoing efforts to further refine the identification, definition, and measurement of the constellation of characteristics important to different specializations of explosive detection tasks. Standardization of traditional working dog assessment techniques, incorporation of elements of proven behavioral questionnaires, and continuing evolution of the use of cognitive measures promise to advance selection technology. Finally, accurate identification and validation of selection measures will rely on continual feedback on dogs' operational performance post-selection.

## Author Contributions

LL and LW wrote the first draft of the manuscript. BR developed concepts and contributed content. MS created Figure 1. SK, MS, PH, and CA contributed to the development of the paper and revised content. All authors read and approved the final draft.

## Conflict of Interest

The authors declare that the research was conducted in the absence of any commercial or financial relationships that could be construed as a potential conflict of interest.
